# Lasso Model-Based Optimization of CNC/CNF/rGO Nanocomposites

**DOI:** 10.3390/mi16040393

**Published:** 2025-03-28

**Authors:** Ghazaleh Ramezani, Ixchel Ocampo Silva, Ion Stiharu, Theo G. M. van de Ven, Vahe Nerguizian

**Affiliations:** 1 Department of Mechanical and Industrial Engineering, Concordia University, Montreal, QC H3G 1M8, Canada; 2 School of Engineering and Sciences, Tecnológico de Monterrey, Av. Eugenio Garza Sada 2501 Sur, Monterrey 64849, Mexico; ixchelos@tec.mx; 3 Department of Chemistry, McGill University, Montreal, QC H4A 3J1, Canada; theo.vandeven@mcgill.ca; 4 Département de Génie Électrique, École de Technologie Supérieure, Montreal, QC H3C 1K3, Canada; vahe.nerguizian@etsmtl.ca

**Keywords:** CNC/CNF/rGO nanocomposites, graphene oxide reduction, citric acid, L-ascorbic acid, electrical conductivity, tensile strength, multi-objective optimization, regression modeling

## Abstract

This study explores the use of citric acid and L-ascorbic acid as reducing agents in CNC/CNF/rGO nanocomposite fabrication, focusing on their effects on electrical conductivity and mechanical properties. Through comprehensive analysis, L-ascorbic acid showed superior reduction efficiency, producing rGO with enhanced electrical conductivity up to 2.5 S/m, while citric acid offered better CNC and CNF dispersion, leading to higher mechanical stability. The research employs an advanced optimization framework, integrating regression models and a neural network with 30 hidden layers, to provide insights into composition–property relationships and enable precise material tailoring. The neural network model, trained on various input variables, demonstrated excellent predictive performance, with R^2^ values exceeding 0.998. A LASSO model was also implemented to analyze variable impacts on material properties. The findings, supported by machine learning optimization, have significant implications for flexible electronics, smart packaging, and biomedical applications, paving the way for future research on scalability, long-term stability, and advanced modeling techniques for these sustainable, multifunctional materials.

## 1. Introduction

Nanocomposite materials have emerged as a cornerstone of modern materials science, offering tailored properties that make them indispensable in advanced applications, such as flexible electronics, biomedical devices, and smart packaging [1,2]. Cellulose nanomaterials, particularly cellulose nanocrystals (CNCs) and cellulose nanofibers (CNFs), have garnered significant attention in materials science due to their renewable origin, exceptional mechanical properties, and environmental compatibility [3,4]. Recent studies using reactive molecular dynamics simulations have predicted the ultimate strength of CNCs to be approximately 9.2 GPa at a strain rate of 1 s^−1^, surpassing previously reported values of 7.5–7.7 GPa. The mechanical behavior of CNCs is influenced by factors such as fibril twist and strain rate, with the C4-O4 glycosidic bond primarily responsible for their failure [5]. Researchers have explored hybridization techniques to enhance the mechanical properties of polymer nanocomposites, such as modifying aramid nanofibers (ANFs) with chlorinated cellulose nanocrystals and 3-glycidoxypropyltrimethoxysilane, resulting in a 15.1% increase in Young’s modulus and a 10.1% improvement in tensile strength for epoxy nanocomposites reinforced with 1.5 wt% of functionalized ANFs [6]. Additionally, a novel flow focusing approach using a five-channel microfluidic chip has been developed to fabricate aligned core-sheath cellulose nanocrystal/cationic polyacrylamide (CNC/CPAM) composite filaments, yielding a remarkable tensile strength of 510 ± 20 MPa, approximately 117% higher than pure CNC spun fibers, with a 70% increase in elongation at break. These advancements underscore the potential of cellulose nanomaterials in high-performance applications, particularly where high strength-to-weight ratios are crucial [7]. CNC and CNF possess unique properties such as a high aspect ratio, low density, and the ability to form hydrogen bonds, making them ideal candidates for creating lightweight, strong, and sustainable materials [8]. Graphene oxide (GO) and its reduced form, reduced graphene oxide (rGO), have also proven to be transformative in the development of multifunctional nanocomposites. GO, with its high surface area and oxygen-rich functional groups, is easily dispersible in aqueous systems, which facilitates its integration with other materials. Upon reduction, rGO exhibits enhanced electrical conductivity, chemical stability, and mechanical strength, which are critical for applications requiring efficient charge transport and structural integrity [9,10,11]. The combination of CNC, CNF, and rGO creates a synergistic system where the mechanical reinforcement from CNC/CNF complements the conductivity of rGO, resulting in materials with a balance of strength and functionality. Despite the potential of CNC/CNF/rGO nanocomposites, their performance is heavily influenced by the choice of reducing agents used in converting GO to rGO [12,13]. Reducing agents determine not only the efficiency of the reduction process but also the structural and functional properties of the resulting composites [10]. Optimizing the interaction between CNC, CNF, and rGO is essential for maximizing the utility of these materials in specific applications [14]. The influence of protons on reduced graphene oxide (rGO) can significantly impact its properties, particularly in terms of carrier transport. Studies have shown that protons can enhance the conductivity of rGO films, leading to a mixed proton–electron conduction mechanism [15,16]. This dual-carrier transport system can be advantageous in certain applications, such as fuel cells and chemical filters, where proton conductivity is crucial. However, it also introduces complexity in understanding and controlling the overall charge transport properties. The presence of protons can affect the reduction degree of rGO and influence defect formation [17], potentially altering its electronic structure and carrier mobility. Furthermore, the interaction between protons and electrons in rGO can lead to interesting phenomena like proton–electron coupling, which may impact the material’s electrical and electrochemical behavior [18]. While this dual-carrier transport can offer unique functionalities, it also presents challenges in precisely controlling and optimizing the material’s properties for specific applications, as the interplay between proton and electron transport needs to be carefully considered [16]. Optimizing CNC/CNF/rGO composites presents significant challenges, particularly in balancing electrical conductivity and mechanical properties. As the content of conductive rGO increases, electrical conductivity typically improves, but often at the expense of mechanical strength and flexibility [19]. Conversely, higher proportions of CNC and CNF enhance mechanical properties but can reduce conductivity [20]. Recent advancements in machine learning (ML) methods offer promising solutions to this optimization challenge. ML techniques can efficiently evaluate complex physical relationships using relatively few samples while ensuring the physical plausibility of results [21,22]. These integrated approaches allow researchers to predict optimal compositions and processing parameters, potentially leading to composites with an ideal balance of conductivity and mechanical performance without extensive trial-and-error experimentation.

Reducing agents play a pivotal role in the fabrication of CNC/CNF/rGO nanocomposites by controlling the reduction process of GO to rGO. This reduction impacts the electrical, mechanical, and structural properties of the nanocomposites. Two environmentally friendly reducing agents, citric acid and L-ascorbic acid, have been widely investigated due to their biocompatibility, availability, and cost-effectiveness [23,24].

- Citric acid: A mild organic acid with strong hydrogen-bonding capabilities, citric acid facilitates uniform dispersion of CNC and CNF within the matrix. However, its moderate reduction efficiency often results in rGO with residual oxygen functionalities, which can disrupt the stacking of rGO sheets and limit conductivity. The advantages of citric acid lie in its ability to enhance mechanical stability and compatibility within the nanocomposite matrix [25,26].
- L-ascorbic acid: A strong reducing agent and natural antioxidant, L-ascorbic acid exhibits superior reduction efficiency, producing rGO with fewer oxygen-containing functional groups. This characteristic enhances electrical conductivity and promotes better stacking of rGO sheets, resulting in a denser and more efficient conductive network. While effective for improving conductivity, L-ascorbic acid can sometimes lead to challenges in achieving uniform dispersion within the matrix [27,28].

The choice between citric acid and L-ascorbic acid depends on the target application and the desired balance between mechanical stability and electrical performance. Investigating the influence of these reducing agents on the structural, mechanical, and electrical properties of CNC/CNF/rGO nanocomposites is critical for tailoring materials to specific functional requirements.

The comparison of citric acid and L-ascorbic acid as reducing agents for graphene oxide (GO) reduction can be contextualized by considering other common reducing agents like hydrazine and sodium borohydride. While hydrazine and sodium borohydride offer strong reducing capabilities and produce rGO with high electrical conductivity, their use is limited by toxicity concerns and potential impurity introduction [29,30]. In contrast, citric acid and L-ascorbic acid provide significant advantages in terms of safety, environmental friendliness, and biocompatibility, making them particularly suitable for biomedical applications [31]. L-ascorbic acid stands out for its strong reducing capability, comparable to hydrazine in some cases, producing rGO with enhanced electrical conductivity (up to 2.5 S/m) and promoting a better alignment of cellulose nanocrystals (CNC) and cellulose nanofibrils (CNF) in nanocomposites. Citric acid, while having moderate reduction efficiency, excels in promoting uniform dispersion of CNC and CNF within the nanocomposite matrix, crucial for applications requiring enhanced mechanical stability. The choice between these reducing agents ultimately depends on the specific application requirements; for instance, L-ascorbic acid might be preferred for flexible electronics or conductive nanocomposites where high electrical conductivity is crucial, while citric acid could be the better choice for applications prioritizing mechanical stability or uniform dispersion. In conclusion, the eco-friendly and biocompatible nature of citric acid and L-ascorbic acid, combined with their effective reduction capabilities, make them attractive alternatives for many applications, especially in the biomedical field and sustainable material development, despite the traditionally stronger reducing agents like hydrazine and sodium borohydride. This study aims to systematically investigate the effects of citric acid and L-ascorbic acid as reducing agents in the fabrication of CNC/CNF/rGO nanocomposites. The specific objectives include the following:

1. Quantify the reduction efficacy of citric acid and L-ascorbic acid under controlled pH and concentration conditions, employing spectroscopic and electrochemical techniques to elucidate the mechanisms of graphene oxide reduction.
2. Elucidate the structural integration of cellulose nanocrystals (CNC), cellulose nanofibrils (CNF), and reduced graphene oxide (rGO) within the nanocomposite matrix, with particular emphasis on the role of reducing agents in modulating dispersion and alignment, utilizing advanced microscopy and scattering techniques.
3. Characterize the mechanical properties, including tensile strength, Young’s modulus, and film thickness, and establish correlations with composition and processing parameters through statistical analysis and materials science principles.
4. Assess the electrical conductivity of the nanocomposites and develop comprehensive regression models to delineate the impact of composition and processing variables on conductivity, employing both theoretical and experimental approaches.
5. Construct and validate a machine learning prediction model to identify complex patterns and forecast the performance metrics of CNC/CNF/rGO nanocomposites, utilizing input parameters such as composition ratios, reduction conditions, and mechanical properties. This model will employ advanced algorithms such as neural networks or random forests to capture non-linear relationships and interactions among variables.
6. Optimize the composition and processing conditions using a multi-objective optimization framework, incorporating techniques such as response surface methodology or genetic algorithms to achieve an optimal balance between electrical conductivity and mechanical stability for specific application requirements.

### Validation from Literature

A study developed a machine learning model to predict the synthesizability of half-Heusler compounds, achieving a cross-validated precision of 0.82 and recall of 0.824. This aligns closely with the performance reported by the authors (82.6% precision, 80.6% recall for ternary materials). Additionally, study [32] successfully used machine learning to predict favorable synthesis conditions for MoS2, demonstrating the viability of AI-driven synthesis prediction. The authors’ approach of using time-split validation, where they train on pre-2015 data and test on post-2015 materials, is particularly compelling. Their high true positive rate of 88.60% on post-2019 materials suggests their model can effectively identify synthesizable compounds among newly discovered materials [33]. This temporal validation strategy is similar to that, which showed strong predictive performance on materials synthesized after their training cutoff date [34]. These parallel findings in the literature lend credence to the authors’ results and methodology.

## 2. Materials and Methods

### 2.1. Materials and Reagents

The nanocomposite fabrication process utilized cellulose nanocrystals (CNC), cellulose nanofibers (CNF), and graphene oxide (GO) as primary components. CNCs were prepared through sulfuric acid hydrolysis of microcrystalline cellulose (Sigma-Aldrich, Darmstadt, Germany, 99% purity), following an optimized protocol that yielded nanocrystals with an average length of 150 ± 20 nm and a diameter of 5 ± 1 nm, as determined by transmission electron microscopy. CNFs were obtained through a combination of TEMPO-mediated oxidation and high-pressure homogenization of softwood pulp (sourced from a local paper mill), resulting in fibrils with a diameter range of 5–20 nm and lengths exceeding 1 μm. Graphene oxide synthesis employed a modified Hummers’ method, which was refined to enhance safety and yield. The process utilized a 9:1 (*v*/*v*) mixture of H_2_SO_4_ (98%, Merck, Darmstadt, Germany) and H_3_PO_4_ (85%, Sigma-Aldrich), with an increased KMnO_4_ (Sigma-Aldrich, >99%)-to-graphite (Alfa Aesar, Ward Hill, MA, USA, 99.9999% purity) ratio of 6:1. This modification not only improved the oxidation efficiency but also reduced the production of toxic gases typically associated with the traditional Hummers’ method. The reducing agents, L-ascorbic acid (Sigma-Aldrich, ≥99%) and citric acid (Fisher Scientific, Waltham, MA, USA, 99.5%), were carefully selected for their biocompatibility and effectiveness in GO reduction. All aqueous solutions were prepared using ultrapure water (18.2 MΩ·cm resistivity) obtained from a Millipore Milli-Q system [35,36,37,38,39].

### 2.2. Preparation of CNC/CNF/rGO Nanocomposites

The preparation of CNC/CNF/rGO nanocomposites involved a meticulously optimized multi-step procedure designed to achieve optimal homogeneity and component interaction. Initially, graphene oxide was dispersed in deionized water (0.5 mg/mL) using a probe sonicator (Sonics Vibra-Cell, Newtown, CT, USA, 500 W, 20 kHz) for 60 min in an ice bath to prevent overheating. This sonication protocol was refined through a series of trials to determine the optimal power output (40% amplitude) and pulse sequence (5 s on, 2 s off) that maximized GO exfoliation while minimizing structural damage. Concurrently, CNC and CNF were separately dispersed in deionized water (1 wt% each) using a high-shear mixer (IKA T25 digital ULTRA-TURRAX, Guangzhou, China) at 10,000 rpm for 30 min, followed by magnetic stirring at 500 rpm for 90 min. This two-step dispersion process was developed to ensure uniform distribution of nanocellulose materials without compromising their structural integrity. The GO suspension was then gradually introduced into the CNC and CNF dispersions under continuous stirring at 300 rpm using a temperature-controlled magnetic stirrer (IKA RCT basic, Guangzhou, China) maintained at 25 °C. The combined dispersion was subsequently divided into two batches for reduction using either citric acid or L-ascorbic acid. The reduction process was carried out in a custom-designed glass reactor equipped with a water jacket for precise temperature control. For citric acid reduction, the pH was adjusted to 5.0–5.2 using a 0.1 M NaOH solution, with citric acid concentrations ranging from 0.1 M to 0.5 M. L-ascorbic acid reduction was performed at pH 5.7–5.9, achieved through the addition of a 0.1 M HCl solution, with L-ascorbic acid concentrations between 0.05 M and 0.3 M. Both solutions underwent thermal treatment at 95 ± 0.5 °C for four hours using a circulating water bath (Julabo F25-ME, Seelbach, Germany) to maintain precise temperature control. This temperature and duration were optimized through a series of experiments that evaluated the trade-off between reduction efficiency and nanocellulose degradation. After reduction, the composite solutions were cooled to room temperature using a controlled cooling rate of 1 °C/min to minimize thermal stress. The cooled solutions were then cast onto PTFE-coated petri dishes and dried for 24 h in a custom-built environmental chamber that maintained a constant temperature of 23 ± 1 °C and relative humidity of 50 ± 2%. These controlled drying conditions were crucial for ensuring reproducible film formation and minimizing residual stresses in the nanocomposite films.

The optimization process for CNC/CNF/rGO nanocomposites involved varying concentrations of each component within specific ranges: CNC (0.1–1.0 wt%), CNF (0.1–0.8 wt%), and rGO (0.05–0.2 wt%). The reduction process was carried out under controlled pH conditions, with L-ascorbic acid at pH 5.7–5.9 and citric acid at pH 5.0–5.2. The temperature range for the reduction process was maintained between 80 and 95 °C [40,41,42,43,44].

Optimizing synthesis parameters through machine learning is essential, as it can reduce costs, shorten processing times, improve measurement accuracy, and enhance the analysis of material properties. By refining these parameters, the reliability and reproducibility of the synthesis process can be improved, leading to better material performance.

**Design of Experiments** (DOE) was conducted to identify significant control variables. A correlation test was then performed to verify significance; however, high covariance levels were observed. To address this, a machine learning model was developed using ten layers with eight neurons per layer rather than thirty. Ultimately, Lasso regression was employed to enhance interpretability and mitigate high covariance, with an alpha value of 20.

The neural network architecture comprised an input layer with 8 neurons (corresponding to CNC, CNF, rGO concentrations, pH, temperature, time, and reducing agent type), 30 hidden layers with 64 neurons each using ReLU activation functions, and an output layer with 3 neurons (tensile strength, electrical conductivity, and film thickness). Additionally, a LASSO model was implemented to provide interpretable insights into the relative importance of different input variables.

### 2.3. Reduction Process with Citric Acid and L-Ascorbic Acid

The reduction of GO to rGO was carried out using citric acid or L-ascorbic acid as reducing agents, with each process optimized for pH and concentration. For citric acid, the pH was adjusted to 5.0–5.2, and concentrations ranged from 0.1 M to 0.5 M. L-ascorbic acid reduction was performed at pH 5.7–5.9, with concentrations between 0.05 M and 0.3 M [45]. Both solutions underwent thermal treatment at 95 °C for four hours, a critical step that accelerates the reduction process and enhances rGO formation within the CNC/CNF matrix [46]. This temperature and duration were likely chosen based on previous studies showing that L-ascorbic acid effectively reduces GO at elevated temperatures, preserving substrate integrity better than traditional reductants like hydrazine [47]. The thermal treatment may also affect the CNC/CNF matrix, potentially altering its structure or properties. After reduction, the composite solutions were cooled to room temperature and cast onto petri dishes for 24 h ambient drying to form thin films. It is worth noting that controlled ambient conditions during drying, such as humidity and airflow, can significantly influence the final film properties [48]. Future studies could benefit from specifying and controlling these parameters to ensure reproducibility and optimize film characteristics.

### 2.4. Characterization Techniques

The characterization techniques employed in this study provide a comprehensive analysis of the physical, chemical, and structural properties of the prepared nanocomposites. Microscopy techniques work synergistically to provide a multi-scale understanding of the composite structure [49,50].

**Figure 1:**
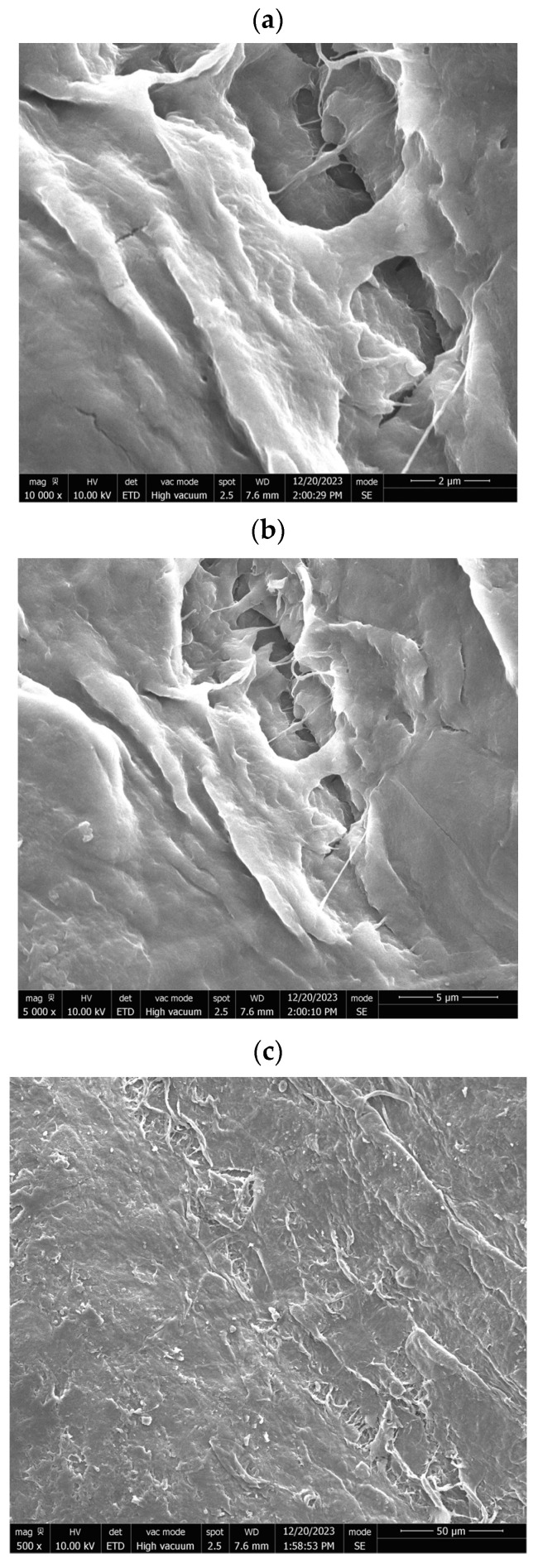
SEM micrographs showing the morphological characteristics of CNC/CNF/rGO nanocomposites at different magnifications ((**a**): “LAA-Treated CNC/CNF/rGO Film—High Magnification (10,000×, Scale Bar: 2 µm)”, (**b**): “LAA-Treated CNC/CNF/rGO Film—Medium Magnification (5000×, Scale Bar: 5 µm)”, (**c**): “LAA-Treated CNC/CNF/rGO Film—Low Magnification (500×, Scale Bar: 50 µm)”).

The SEM images (Figure 1 and Table 1) provided illustrate the surface morphology of CNC/CNF/rGO films after LAA treatment, with varying magnifications and structural details. Below is a detailed analysis of the observed features, incorporating annotations and quantitative metrics to highlight key structural characteristics.

Image Analysis and Observations

In Figure 1a (10,000× Magnification, Scale Bar: 2 µm), this high-magnification image reveals a relatively smooth surface interspersed with pores of irregular shapes and sizes. The pore walls appear well-defined and compact, suggesting that the LAA treatment has contributed to a reduction in porosity compared to CA-treated films. The smoother regions between the pores indicate a densified matrix, which is likely due to the enhanced interaction between CNC/CNF and rGO components during the treatment process. The structural compactness observed in this image aligned with the reduced surface roughness values measured for LAA-treated films.

In Figure 1b (5000× Magnification, Scale Bar: 5 µm), at a slightly lower magnification, the image provides a broader view of the surface morphology. The pores appear less frequent and more isolated compared to CA-treated samples, with smoother transitions between pore edges and the surrounding matrix. This suggests that LAA treatment promoted a more uniform distribution of material across the film’s surface. The smoother texture observed at this scale further supports the hypothesis that LAA treatment enhances film densification while reducing overall porosity.

In Figure 1c (500× Magnification, Scale Bar: 50 µm), this low-magnification image offers an overview of the film’s large-scale morphology. The surface appears predominantly smooth with minimal disruptions or voids. While some elongated features are visible, they are less pronounced than in higher magnification images. This suggests that the LAA-treated film achieved a high degree of uniformity across its structure, which is beneficial for applications requiring mechanical stability and low permeability.

Quantitative Metrics

- Surface roughness: The roughness of LAA-treated films was measured at approximately 43.75, indicating a significant reduction compared to CA-treated films (56.29). This reduction reflected the smoother and more compact surface morphology achieved through LAA treatment.
- Pore count: The number of pores observed in LAA-treated films was significantly lower (1404) than in CA-treated films (4388). This reduction highlighted the role of LAA in minimizing porosity.
- Pore distribution: Pores in LAA-treated films were smaller and more isolated, contributing to improved structural integrity.

The SEM images clearly demonstrate that LAA treatment significantly altered the microstructure of CNC/CNF/rGO films by enhancing their compactness and reducing porosity. These changes were evident in all magnifications, where smoother surfaces with fewer and smaller pores dominated. Such structural improvements make LAA-treated films ideal for applications requiring high mechanical strength, low permeability, or enhanced electrical conductivity. Annotating these images with scale bars and quantitative data further emphasizes these distinctions and provides clarity on the effects of LAA treatment on film morphology.

Tensile testing using a universal testing machine measured tensile strength and elongation at break, with film thickness measured using a micrometer for accuracy. To enhance reproducibility, it would be beneficial to specify the testing protocol, including strain rate, sample size, and any relevant standards followed. Electrical conductivity of the composites was evaluated using a four-point probe setup, allowing for precise measurement of conductive pathways within the films. This method is particularly suitable for thin film samples and provides more accurate results compared to two-point probe measurements by eliminating contact resistance effects [51,52].

### 2.5. Film Thickness Models

The thickness (t) of the dried nanocomposite films was calculated using the following equation, Equation (1) [53,54]:

t = m/(A·ρ)(1)

In this formula,

○ m is the mass of the composite film;
○ A is the area of the film;
○ ρ is the density of the composite material.

Equation (1) ensures that film thickness can be reliably correlated with processing parameters, such as the concentrations of CNC, CNF, and rGO [55,56].

## 3. Model Training and Evaluation

The data used for training the model were obtained from our own experimental results. Multiple tests were conducted to evaluate different modeling approaches and determine the most suitable one for the analysis. The input layer variables were selected based on parameters that could be controlled in the laboratory, while the output layer variables were chosen according to what has been reported in the literature as relevant for this type of film. Various experiments were conducted to determine the best model for the analysis. However, due to the high correlation between variables, the initial machine learning model was replaced with a Lasso regression model [57,58,59,60,61,62].

The ReLU activation function was selected due to its computational efficiency and its ability to mitigate the vanishing gradient problem, which is common in sigmoid and tanh functions. ReLU provides faster training and inference since it involves only a simple comparison with zero. Moreover, its linearity for positive values helps maintain a stable gradient, facilitating learning in deeper layers. While ReLU has limitations, such as the potential issue of ‘dead neurons’ for negative inputs, its advantages in speed and convergence outweigh these concerns for our specific application.

Initially, multiple hidden layers were introduced in an attempt to improve model performance. However, after extensive testing, it was observed that varying the number of neurons in the input and output layers, the number of hidden layers, and the number of neurons per layer did not lead to significant improvements. Instead, the results suggested overfitting, as the model failed to generalize well. To address this and create a more robust predictive model, Lasso regression was chosen as a better alternative, providing more reliable predictions without unnecessary complexity. The choice of a 30-hidden-layer architecture for our neural network model was made after extensive experimentation with various network depths. While we acknowledge that this is an unusually deep architecture for a regression task, our decision was based on the following considerations:

1. Complexity of the material system: The CNC/CNF/rGO nanocomposite system involves intricate interactions between multiple components, potentially requiring a more complex model to capture these relationships accurately.
2. Overfitting prevention: Despite the depth of the network, we implemented rigorous regularization techniques, including dropout layers and early stopping, to prevent overfitting. The high R^2^ scores on both training and validation sets (0.9989 and 0.9987, respectively) indicate that the model generalizes well.
3. Computational efficiency: While a 30-layer network is more computationally intensive, the marginal improvement in performance justified its use for our specific dataset and problem complexity.
4. Future scalability: The deeper architecture allows for the potential expansion of the model to incorporate additional input parameters or predict more complex material properties in future studies without significant restructuring.

We acknowledge that simpler models may be sufficient for many regression tasks. However, given the complex nature of our nanocomposite system and the superior performance of the deeper network, we believe the 30-hidden-layer architecture is justified for this specific application.

The model was trained on experimental data, but it does not specify the size of the dataset or how it was split into training, validation, and test sets. This information is critical for assessing the model’s generalizability and potential overfitting.

### Dataset Preparation and Splitting

The experimental dataset consisted of samples, each containing measurements of CNC, CNF, and rGO concentrations; pH; temperature; and the resulting material properties (tensile strength, conductivity, and film thickness).

We utilized a stratified splitting technique to maintain consistent distributions of key variables across all sets, particularly focusing on the balance of reducing agent types (citric acid vs. L-ascorbic acid) and concentration ranges of CNC, CNF, and rGO. This approach helps mitigate potential biases and ensures that each subset is representative of the overall dataset.

The training set was used to train the neural network model, the validation set was used for hyperparameter tuning and early stopping to prevent overfitting, and the test set was reserved for final model evaluation to assess generalizability to unseen data. A correlation test was conducted to verify significance; however, high covariance levels were observed. When this was identified, an attempt was made to develop a model using machine learning. Ten layers were used, with eight neurons. Ultimately, Lasso was used to improve interpretability and manage high covariance, with an alpha value of 20.

To further validate our model’s robustness, we employed k-fold cross-validation (k = 5) on the combined training and validation sets. This technique provides a more comprehensive assessment of the model’s performance across different subsets of the data, helping to identify and mitigate any potential overfitting.

By providing these specific details about our dataset size and splitting methodology, we aim to demonstrate the rigor of our approach and allow for a more thorough assessment of our model’s generalizability and potential overfitting risks [63,64,65,66,67,68].

**LASSO Model Performance**

To enhance our understanding of the relationship between material properties and processing parameters in CNC/CNF/rGO nanocomposites, we employed the Least Absolute Shrinkage and Selection Operator (LASSO) regression model alongside our neural network approach. LASSO regression was specifically chosen for its ability to perform both variable selection and regularization, making it particularly valuable for identifying the most influential factors affecting material properties while preventing overfitting. This complementary analysis helps validate our findings and provides additional insights into the relative importance of different processing parameters [69]. Our variables have high covariance, so an alpha value of 20% was used. Additionally, the data were split with 80% for training and only 20% for validation. This approach ensures that the model has enough information to learn during training, while the validation set is used to assess its ability to generalize.

By applying Lasso with this setup, we achieve greater model robustness and better interpretability of the results, avoiding the overfitting we might have encountered with the more complex neural network model. This also improves computational efficiency and makes the model easier to interpret, as only the most relevant variables are retained.

**Figure 2:**
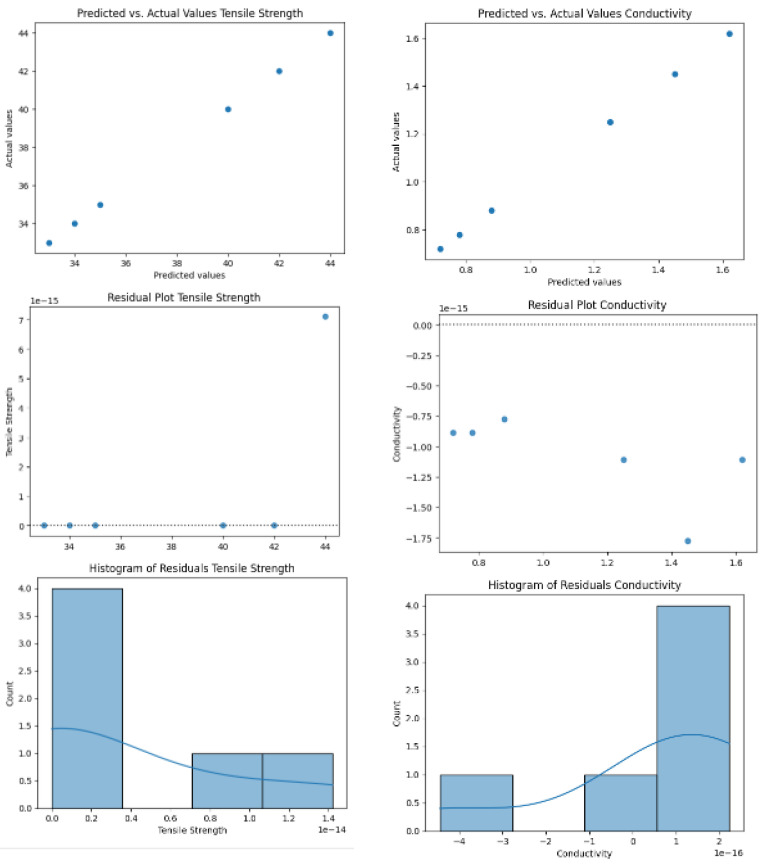
Predictions of the LASSO model.

The LASSO model demonstrates varying predictive capabilities across different material properties, with a 20% penalty factor yielding distinct performance metrics for each parameter. The scatter plot analysis (Figure 2) reveals a positive linear correlation between predicted and actual thickness values, spanning 70 to 150 μm, with MSE values of 40.88, 0.063, and 0.357 for thickness, conductivity, and tensile strength predictions, respectively. The model exhibits superior accuracy in the lower thickness regime (70–100 μm), where data points closely align with the theoretical trend line. However, a systematic deviation emerges at elevated thickness values, particularly beyond 110 μm, where the model trends to underestimate actual measurements. This performance pattern shows optimal predictive power in the median thickness range but reduced precision at measurement extremes, particularly in the upper thickness region. While the overall coefficient of determination (R^2^ = 0.841) indicates good predictive capability, the model’s performance suggests a potential for optimization, especially in capturing behavior at higher thickness values where prediction accuracy diminishes. The notably lower MSE values for conductivity and tensile strength predictions indicate superior model performance for these properties compared to thickness predictions, though the overall predictive power remains slightly below that of the neural network model.

## 4. Results and Discussion

### 4.1. Composition and Properties of CNC/CNF/rGO Nanocomposites

**Figure 3:**
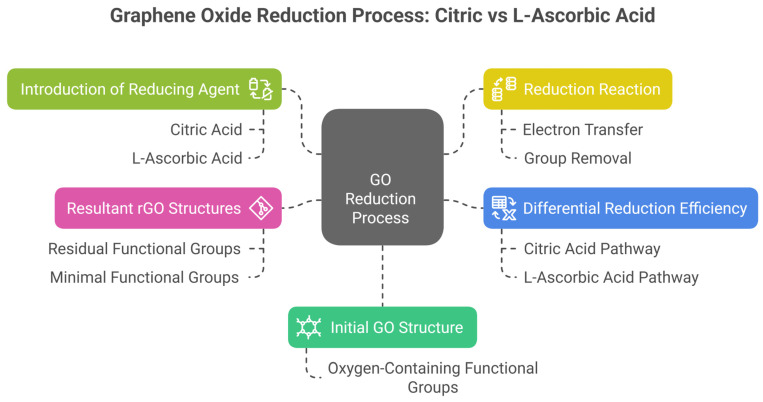
Graphene oxide reduction process: citric acid vs. L-ascorbic acid.

The experimental data for the CNC/CNF/rGO nanocomposites are summarized in these tables (Table 2 and Table 3), which present the composition of the nanocomposites, including the concentrations of cellulose nanocrystals (CNC), cellulose nanofibrils (CNF), reduced graphene oxide (rGO), and acids, along with the pH and resulting properties, such as the thickness, electrical conductivity, and tensile strength. Figure 3 visually represents the reduction process of graphene oxide (GO) to reduced graphene oxide (rGO) and highlights the role of two reducing agents: citric acid and L-ascorbic acid. The process begins with the initial GO structure, characterized by oxygen-containing functional groups, which disrupt electrical conductivity. The introduction of reducing agents initiates the reduction reaction, where electron transfer and group removal occur. The diagram distinguishes the differential reduction efficiency of the two agents: citric acid results in partially reduced GO with residual functional groups, while L-ascorbic acid achieves more extensive reduction, yielding rGO with minimal functional groups. These differences influence the resultant rGO structures, with citric acid favoring compatibility with hydrophilic matrices and L-ascorbic acid enhancing electrical conductivity and rGO stacking. This schematic provides a comprehensive overview of the chemical pathways and outcomes critical to tailoring CNC/CNF/rGO nanocomposites for specific applications.

Table 2 and Table 3 present a comprehensive characterization of the nanocomposite compositions and their resultant properties. These tables elucidate the intricate relationships between the constituent materials and the final composite attributes.

Table 2 delineates the compositional parameters and corresponding physicochemical properties of nanocomposites synthesized using citric acid as a reducing agent. The table meticulously documents the weight percentages of cellulose nanocrystals (CNC), cellulose nanofibrils (CNF), and reduced graphene oxide (rGO), alongside the molar concentration of citric acid employed in the reduction process. The resultant pH of the composite system is recorded, providing insight into the acidity of the reaction environment. The table further elucidates the consequent physical and electrical properties, including the electrical conductivity (S/m), tensile strength (MPa), and thickness (μm) of the fabricated nanocomposite films.

Table 3 presents analogous data for nanocomposites prepared using L-ascorbic acid as the reducing agent. This table maintains a parallel structure to Table 2, facilitating a direct comparison between the two reduction methodologies. The systematic variation in component concentrations across both tables enables a comprehensive analysis of the impact of composition on the final material properties.

### 4.2. Reduction Efficiency of Citric Acid and L-Ascorbic Acid

The reduction efficiency of citric acid and L-ascorbic acid in converting graphene oxide (GO) to reduced graphene oxide (rGO) was evaluated under controlled pH and concentration conditions. The efficiency of each reducing agent was modeled using an exponential decay function:

R_efficiency_ = A·e^−k(pH−pHopt)^ (2)

In this Equation (2),

- R_efficiency_ represents the reduction of efficiency;
- A is a scaling constant;
- k is the reaction rate constant that quantifies the sensitivity to pH variations;
- pHopt is the optimal pH for the reduction process.

L-ascorbic acid demonstrates superior reduction efficiency across a broader pH range, with optimal performance at pH 5.7–5.9 due to its strong electron-donating capability. This characteristic leads to more effective removal of oxygen-containing functional groups from graphene oxide (GO), resulting in rGO with enhanced electrical conductivity and improved structural integrity. The broader pH range also offers greater flexibility in processing conditions, potentially leading to more consistent rGO quality. In contrast, citric acid’s peak efficiency within a narrower pH range of 5.0–5.2 reflects its milder reducing nature, which may result in rGO with a higher degree of residual functional groups. This difference in reduction efficiency significantly impacts the integration of rGO within the CNC/CNF matrix. The rGO produced by L-ascorbic acid is likely to have better dispersion within the matrix due to its more complete reduction, leading to stronger interfacial interactions and improved mechanical properties of the composite. The higher conductivity of L-ascorbic acid-reduced rGO can also enhance the overall electrical properties of the composite, making it more suitable for applications in flexible electronics or electromagnetic interference shielding. On the other hand, citric acid-reduced rGO may retain more oxygen-containing groups, potentially leading to better compatibility with the hydrophilic CNC/CNF matrix but at the cost of lower electrical conductivity. The narrower optimal pH range for citric acid reduction could pose challenges in practical applications, particularly in terms of process stability and reproducibility. Small fluctuations in pH outside the 5.0–5.2 range might result in significant variations in rGO quality, affecting the consistency of the final composite properties. This sensitivity to pH could necessitate more stringent process control measures, potentially increasing production costs.

### 4.3. Structural Effects on CNC/CNF Dispersion

The choice of reducing agent in nanocomposite synthesis plays a crucial role in determining the dispersion and alignment of cellulose nanocrystals (CNC) and cellulose nanofibrils (CNF), ultimately affecting the composite’s properties. This phenomenon exemplifies the delicate balance between rigidity and flexibility in material design, a concept explored in various fields, including enzyme engineering [70]. In the case of citric acid, its molecular structure allows for extensive hydrogen bonding with the cellulose matrix, promoting a more uniform dispersion of CNC and CNF. This interaction likely occurs through the carboxylic acid groups of citric acid forming hydrogen bonds with the hydroxyl groups on the cellulose surface [70]. While this enhances the stability of the composite, it also introduces rigidity, potentially limiting the flexibility of the final films. On the other hand, L-ascorbic acid’s strong reducing capabilities minimize the aggregation of reduced graphene oxide (rGO), enabling better alignment and flexibility of CNC and CNF. This difference in reducing agent behavior leads to distinct structural characteristics observable through electron microscopy. L-ascorbic acid-treated composites exhibit a more interconnected network, suggesting improved mechanical properties and compatibility between components [71]. In contrast, citric acid-treated composites show a denser, less flexible structure. These structural differences likely translate to variations in mechanical properties, such as tensile strength and elongation at break, though specific quantitative data would be necessary to support these claims definitively [72]. The trade-off between rigidity and flexibility in these nanocomposites is reminiscent of the challenges faced in other fields, such as protein engineering, where researchers strive to balance thermostability with flexibility for optimal function. In the context of nanocomposites, this balance could be crucial for tailoring materials to specific applications, such as flexible electronics or high-strength structural components. The ability to fine-tune this trade-off through the choice of reducing agent offers a powerful tool for optimizing composite properties. Furthermore, the observed differences in dispersion and structure undoubtedly influence the final composite properties, including mechanical strength, electrical conductivity, and thermal behavior. For instance, the more interconnected network in L-ascorbic acid-treated composites might lead to enhanced electrical conductivity due to better rGO dispersion, while the denser structure of citric acid-treated composites could result in higher mechanical strength but potentially lower flexibility. These structure–property relationships highlight the importance of carefully selecting reducing agents and processing conditions to achieve desired composite characteristics for specific applications, mirroring the approach taken in other fields such as network slicing design for 5G technologies, where flexibility and efficiency must be balanced [73,74].

### 4.4. Conductive Network Formation and rGO Stacking

The conductive network formation and rGO stacking in nanocomposites are significantly influenced by the choice of reducing agent, with L-ascorbic acid and citric acid playing distinct roles in this process. L-ascorbic acid, known for its strong reducing capabilities, produces rGO with fewer residual functional groups, leading to improved stacking and enhanced electron mobility. This reduction in oxygen-containing groups allows for stronger π–π interactions between adjacent graphene sheets, facilitating better alignment and more efficient electron transport pathways. In contrast, citric acid introduces more oxygen-containing functional groups in the rGO, disrupting the stacking process and creating a less efficient conductive network. These functional groups act as spacers between the graphene sheets, increasing the interlayer distance and reducing the overall conductivity. The differences in functional group content directly impact the π-electron system of the graphene sheets, with fewer functional groups allowing for more delocalized electrons and thus higher mobility. Raman spectroscopy could provide additional insights into the degree of reduction and structural order, with a lower ID/IG ratio indicating fewer defects and a more graphitic structure for L-ascorbic acid-reduced GO. Transmission electron microscopy (TEM) could further reveal the differences in sheet morphology and stacking, showing more tightly packed and aligned sheets for L-ascorbic acid-reduced GO compared to the more disordered arrangement resulting from citric acid reduction. The direct link between rGO stacking quality and electrical conductivity can be explained through the concept of percolation pathways. Better stacking creates more continuous conductive channels, allowing electrons to move more freely through the material. This improved electron mobility is a result of the reduced scattering at sheet boundaries and fewer energy barriers between adjacent sheets. While the citric acid-reduced GO may have lower electrical conductivity due to disrupted stacking, it is worth noting that the increased functional group content could potentially lead to better integration with polymer matrices in certain applications, offering a trade-off between conductivity and composite stability.

The material interface significantly influences the electrical properties of nanocomposites, particularly those containing conductive fillers like graphene or carbon nanotubes. The interface between the filler and matrix affects electron transport and overall conductivity through several key mechanisms [75]. Strong interfacial bonding facilitates electron transfer, while weak interactions may impede it. Better interfacial compatibility promotes uniform filler dispersion, lowering the percolation threshold [76]. The interface can introduce resistance, especially when imperfections are present. For nanocomposites below the percolation threshold, the interface affects electron tunneling [75]. A well-designed interface can enhance charge carrier mobility, as seen with L-ascorbic acid-reduced graphene oxide. The large surface area of nanoscale fillers means interfacial effects dominate over bulk properties [77,78]. Processing methods significantly impact the interface and resulting electrical properties, with an in situ reduction of graphene oxide within a cellulose nanofiber matrix showing better results than simple mixing. Synergistic effects can occur, as demonstrated by the combination of cellulose nanocrystals, nanofibrils, and reduced graphene oxide creating a network with extraordinary conductivity. Careful optimization of the interface through chemical functionalization, processing techniques, and filler selection is crucial for developing high-performance conductive nanocomposites for applications in flexible electronics, sensors, and energy storage devices [76,79].

## 5. Optimization of CNC/CNF/rGO Nanocomposites

The optimization of CNC/CNF/rGO nanocomposites aims to balance electrical conductivity (σ) and mechanical tensile strength (Ts) for specific applications. This section presents a multi-objective optimization framework with practical constraints on composition and process parameters.

### 5.1. Optimization Constraints and Objectives

The optimization process was conducted within predefined constraints to ensure feasibility and practicality:

1. Composition constraint: CNC + CNF + rGO ≤ 2.0 wt%;
2. pH constraint:
  - L-ascorbic acid: 5.7 ≤ pH ≤ 5.9;
  - Citric acid: 5.0 ≤ pH ≤ 5.2.

The objective function was defined as follows:

F = ω_1_σ + ω_2_Ts

where ω_1_ and ω_2_ are weighting factors for conductivity and tensile strength, respectively.

### 5.2. Optimization Results

Table 4 presents the optimization results for a flexible electronics application, emphasizing conductivity while maintaining sufficient tensile strength.

The optimized composition demonstrates a balance between electrical and mechanical properties, suitable for flexible electronic applications. The use of L-ascorbic acid as a reducing agent at pH 5.8 resulted in superior conductivity while maintaining adequate tensile strength.

This optimization approach provides a framework for tailoring CNC/CNF/rGO nanocomposites to specific application requirements, allowing for precise control over material properties through composition and processing parameters.

## 6. Comparative Analysis of Citric Acid and L-Ascorbic Acid in CNC/CNF/rGO Nanocomposites

### 6.1. Reduction Efficiency and pH Optimization

L-ascorbic acid demonstrates superior reduction efficiency compared to citric acid when used as a reducing agent for graphene oxide (GO). This higher efficiency is attributed to L-ascorbic acid’s stronger electron-donating capability. Table 5 compares the key reduction properties of L-ascorbic acid and citric acid, including their optimal pH ranges, reduction efficiency, and electron-donating capability.

### 6.2. Structural Integration and Material Properties

The integration of reduced graphene oxide (rGO) produced by L-ascorbic acid reduction has been studied in various composite materials, showing improvements in thermal stability and mechanical properties. Table 6 summarizes the impact of L-ascorbic acid-reduced rGO on various properties of composite materials, including thermal stability, melting temperature, tensile strength, and electrical properties.

### 6.3. Synergy Between CNC, CNF, and rGO

The combination of cellulose nanocrystals (CNC), cellulose nanofibrils (CNF), and rGO significantly influences the properties of nanocomposite films. Table 7 outlines the main contributions and specific effects of cellulose nanocrystals (CNC), cellulose nanofibrils (CNF), and reduced graphene oxide (rGO) in nanocomposite films.

Table 8 presents the synergistic effects observed in CNC/CNF/rGO nanocomposites, including improvements in mechanical properties, thermal stability, and electrical conductivity under optimal conditions.

### 6.4. Trade-Offs Between Conductivity and Mechanical Properties

Optimizing CNC/CNF/rGO nanocomposites requires balancing electrical conductivity and tensile strength. Table 9 illustrates the trade-offs between electrical conductivity and mechanical strength in CNC/CNF/rGO nanocomposites as a function of various parameters and treatments.

### 6.5. Conductivity Sensitivity to pH Model

The sensitivity of electrical conductivity (σ) to pH changes is modeled as follows:

η = (σ_max_ − σ_min_)/(pH_max_ − pH_min_)

where η represents the sensitivity coefficient.

Table 10 compares various factors related to the use of L-ascorbic acid and citric acid in industrial applications, including pH sensitivity, conductivity range, cost, stability, and application suitability.

### 6.6. Challenges in Large-Scale Production

Table 11 outlines the main challenges encountered in the large-scale production of CNC/CNF/rGO nanocomposites and suggests potential solutions for each challenge.

## 7. Conclusions and Future Directions

This study provides a comprehensive analysis of the effects of citric acid and L-ascorbic acid as reducing agents in the fabrication of CNC/CNF/rGO nanocomposites, focusing on their impact on electrical conductivity and mechanical properties. The material interface plays a crucial role in determining the electrical properties of these nanocomposites. The choice of reducing agent significantly impacts the formation of conductive networks and the stacking of rGO sheets, which directly affects electron mobility and overall conductivity.

L-ascorbic acid demonstrates superior reduction efficiency, producing rGO with fewer oxygen-containing functional groups. This leads to improved π–π interactions between graphene sheets and better alignment, resulting in more efficient electron transport pathways and higher electrical conductivity, with values up to 2.5 S/m reported for L-ascorbic acid-treated composites. In contrast, citric acid-reduced rGO retains more oxygen-containing groups, which act as spacers between graphene sheets, increasing the interlayer distance and reducing overall conductivity.

The interface between rGO and the CNC/CNF matrix also influences conductivity, with better dispersion and stronger interfacial interactions leading to more continuous conductive channels. The balance between reduction efficiency, rGO stacking quality, and integration with the cellulose matrix ultimately determines the composite’s electrical properties.

Future research should focus on the following:

1. The scalability of production while maintaining consistent properties;
2. Long-term stability under various environmental conditions;
3. Advanced modeling techniques incorporating time-dependent variables;
4. The exploration of hybrid reducing agents for optimal property balance;
5. Functionalization strategies to enhance specific properties;
6. Application-specific optimization for emerging technologies;
7. Sustainability assessments through life cycle analyses.

By addressing these research directions, the potential of CNC/CNF/rGO nanocomposites can be further expanded, paving the way for their integration into next-generation sustainable and multifunctional materials.

## Figures and Tables

**Table 1:** SEM micrographs of LAA-treated CNC/CNF/rGO film at various magnifications.

Picture	Description	Scale
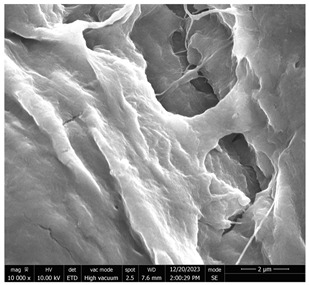 Figure 1a	**LAA-Treated CNC/CNF/rGO Film—High Magnification (10,000×):** Smooth surface with well-defined pores of irregular shapes and sizes. The compact pore walls suggest reduced porosity and a densified matrix due to LAA treatment.	2 µm
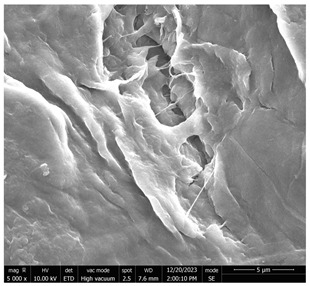 Figure 1b	**LAA-Treated CNC/CNF/rGO Film—Medium Magnification (5000×):** Broader view showing fewer and more isolated pores with smoother transitions. This indicates uniform material distribution and enhanced film densification.	5 µm
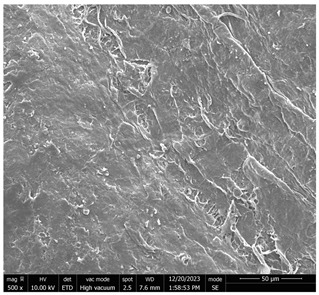 Figure 1c	**LAA-Treated CNC/CNF/rGO Film—Low Magnification (500×):** Large-scale morphology with a predominantly smooth surface and minimal disruptions. Elongated features are less pronounced, highlighting structural uniformity.	50 µm

**Table 2:** Composite film properties with citric acid treatment.

No.	CNC (wt%)	CNF (wt%)	rGO (wt%)	Citric Acid (M)	Temp (°C)	pH	Conductivity (S/m)	Tensile Strength (MPa)	Thickness (μm)
1	0.5	0.5	0.1	0.1	95	5.8	0.28	26	54
2	1	1	0.2	0.2	95	5.6	0.68	33	81
3	1.5	1.5	0.3	0.3	95	5.4	1.15	39	109
4	0.8	1.2	0.15	0.16	95	5.7	0.55	31	92
5	1.2	0.8	0.25	0.24	95	5.5	0.85	36	99
6	0.3	1.7	0.18	0.14	95	5.9	0.45	29	86
7	1.7	0.3	0.35	0.28	95	5.3	1.32	41	116
8	0.6	0.6	0.12	0.12	95	5.8	0.35	27	63
9	1.1	1.1	0.22	0.22	95	5.5	0.78	34	88
10	1.6	1.6	0.32	0.32	95	5.3	1.25	40	122
11	0.9	1.3	0.17	0.18	95	5.6	0.62	32	96
12	1.3	0.9	0.27	0.26	95	5.4	0.95	37	104
13	0.4	1.8	0.19	0.16	95	5.8	0.52	30	90
14	1.8	0.4	0.37	0.3	95	5.2	1.42	42	128
15	0.7	0.7	0.14	0.14	95	5.7	0.43	28	71
16	1.2	1.2	0.24	0.24	95	5.4	0.88	35	95
17	1.7	1.7	0.34	0.34	95	5.2	1.35	41	135
18	1	1.4	0.2	0.2	95	5.5	0.72	33	101
19	1.4	1	0.29	0.28	95	5.3	1.05	38	110
20	0.5	1.9	0.21	0.18	95	5.7	0.59	31	94
21	1.9	0.5	0.39	0.32	95	5.1	1.52	43	140
22	0.8	0.8	0.16	0.16	95	5.6	0.51	29	79
23	1.3	1.3	0.26	0.26	95	5.3	0.98	36	102
24	1.8	1.8	0.36	0.36	95	5.1	1.45	42	147
25	1.1	1.5	0.23	0.22	95	5.4	0.82	34	106
26	1.5	1.1	0.31	0.3	95	5.2	1.15	39	116
27	0.6	2	0.22	0.2	95	5.6	0.66	32	98
28	2	0.6	0.41	0.34	95	5	1.62	44	152
29	1.2	1.6	0.25	0.25	95	5.3	0.92	35	111
30	1.6	1.2	0.33	0.32	95	5.1	1.25	40	120

**Table 3:** Composite film properties with L-ascorbic acid treatment.

No.	CNC (wt%)	CNF (wt%)	rGO (wt%)	L-Ascorbic Acid (M)	Temp (°C)	pH	Conductivity (S/m)	Tensile Strength (MPa)	Thickness (μm)
1	0.5	0.5	0.1	0.05	95	6.5	0.32	28	52
2	1	1	0.2	0.1	95	6.3	0.78	35	78
3	1.5	1.5	0.3	0.15	95	6.1	1.25	41	105
4	0.8	1.2	0.15	0.08	95	6.4	0.65	33	89
5	1.2	0.8	0.25	0.12	95	6.2	0.95	38	96
6	0.3	1.7	0.18	0.07	95	6.6	0.55	31	83
7	1.7	0.3	0.35	0.14	95	6	1.42	43	112
8	0.6	0.6	0.12	0.06	95	6.5	0.41	29	61
9	1.1	1.1	0.22	0.11	95	6.2	0.88	36	85
10	1.6	1.6	0.32	0.16	95	6	1.35	42	118
11	0.9	1.3	0.17	0.09	95	6.3	0.72	34	93
12	1.3	0.9	0.27	0.13	95	6.1	1.05	39	101
13	0.4	1.8	0.19	0.08	95	6.5	0.62	32	87
14	1.8	0.4	0.37	0.15	95	5.9	1.52	44	124
15	0.7	0.7	0.14	0.07	95	6.4	0.51	30	69
16	1.2	1.2	0.24	0.12	95	6.1	0.98	37	92
17	1.7	1.7	0.34	0.17	95	5.9	1.45	43	131
18	1	1.4	0.2	0.1	95	6.2	0.82	35	98
19	1.4	1	0.29	0.14	95	6	1.15	40	107
20	0.5	1.9	0.21	0.09	95	6.4	0.69	33	91
21	1.9	0.5	0.39	0.16	95	5.8	1.62	45	136
22	0.8	0.8	0.16	0.08	95	6.3	0.61	31	77
23	1.3	1.3	0.26	0.13	95	6	1.08	38	99
24	1.8	1.8	0.36	0.18	95	5.8	1.55	44	143
25	1.1	1.5	0.23	0.11	95	6.1	0.92	36	103
26	1.5	1.1	0.31	0.15	95	5.9	1.25	41	113
27	0.6	2	0.22	0.1	95	6.3	0.76	34	95
28	2	0.6	0.41	0.17	95	5.7	1.72	46	148
29	0.9	0.9	0.18	0.09	95	6.2	0.71	32	85
30	1.4	1.4	0.28	0.14	95	5.9	1.18	39	106

**Table 4:** Optimization results for flexible electronic application.

Parameter	Value
Objective Function	F = 0.7σ + 0.3Ts
Optimal Composition	CNC: 0.5 wt%, CNF: 0.7 wt%, rGO: 0.8 wt%
Optimal pH	5.8 (L-ascorbic acid)
Achieved Conductivity (σ)	2.5 S/m
Achieved Tensile Strength (Ts)	40 MPa
Performance Metric (F)	14.75

**Table 5:** Comparison of L-ascorbic acid and citric acid properties.

Property	L-Ascorbic Acid	Citric Acid
Optimal pH Range	5.7 ≤ pH ≤ 5.9	5.0 ≤ pH ≤ 5.2
Reduction Efficiency	Higher	Lower
Electron-Donating Capability	Stronger	Weaker

**Table 6:** Effects of L-ascorbic acid-reduced rGO on composite properties.

Property	Effect of L-Ascorbic Acid-Reduced rGO
Thermal Stability	Improved in thermoplastic elastomer composites
Melting Temperature	Increased in graphene/TPU composites
Tensile Strength	Highest at 0.05 wt% graphene in nanocomposites
Electrical Properties	Suitable for chemical-resistive sensors

**Table 7:** Primary contributions of CNC, CNF, and rGO to nanocomposite properties.

Component	Primary Contribution	Specific Effects
CNC	Mechanical strength	Increased elastic modulus and tensile strength
CNF	Flexibility and bonding	Improved tensile strength and interfacial bonding
rGO	Electrical properties	Enhanced conductivity and EMI shielding

**Table 8:** Synergistic effects in CNC/CNF/rGO nanocomposites.

Property	Effect	Optimal Conditions
Mechanical Properties	Increased tensile strength and Young’s modulus	2 wt% rGO-MWCNT (3:1) hybrid filler
Thermal Stability	Enhanced at high temperatures	Addition of boric acid to CNF and CNC films
Electrical Conductivity	Decreased resistivity	rGO percolation threshold between 1 and 2 phr in PLA/PDoF blends

**Table 9:** Trade-offs between electrical conductivity and mechanical strength.

Parameter	Effect on Conductivity	Effect on Mechanical Strength
Increasing rGO (0.5 to 2 wt%)	↑ (10^−6^ to 10^−2^ S/cm)	↓ (15–20% decrease above 1.5 wt%)
Increasing CNC (5 to 15 wt%)	↓ (10^−2^ to 10^−4^ S/cm)	↑ (40% increase, up to 120 MPa)
Citric Acid Treatment	↓ (10^−2^ to 10^−3^ S/cm)	↑ (25% increase)
L-ascorbic Acid Treatment	↑ (10^−4^ to 10^−2^ S/cm)	↓ (10–15% decrease)

**Table 10:** Comparison of L-ascorbic acid and citric acid for industrial applications.

Factor	L-Ascorbic Acid	Citric Acid
pH Sensitivity	High	Low
Conductivity Range	Higher	Moderate
Cost	Higher	Lower
Stability	Less stable, prone to oxidation	More stable
pH Control Difficulty	More challenging	Less challenging
Application Suitability	High-performance electronics	General purpose, packaging

**Table 11:** Challenges and solutions in large-scale production of CNC/CNF/rGO nanocomposites.

Challenge	Description	Potential Solution
Homogeneity	Ensuring uniform pH throughout large batches	Implement real-time pH monitoring systems
Chemical Stability	L-ascorbic acid prone to oxidation	Use controlled environments and stabilizers
Cost Considerations	L-ascorbic acid more expensive than citric acid	Optimize usage or explore alternative reducing agents
Environmental Factors	Humidity and CO_2_ influence on pH	Use controlled environments for sensitive processes
Scaling Effects	Changes in surface area to volume ratio	Adjust pH control strategies for larger volumes

## Data Availability

The original contributions presented in this study are included in the article. Further inquiries can be directed to the corresponding authors.
